# DLFF-ACP: prediction of ACPs based on deep learning and multi-view features fusion

**DOI:** 10.7717/peerj.11906

**Published:** 2021-08-03

**Authors:** Ruifen Cao, Meng Wang, Yannan Bin, Chunhou Zheng

**Affiliations:** 1 Key Laboratory of Intelligent Computing and Signal Processing of Ministry of Education, School of Computer Science and Technology, Anhui University, Hefei, Anhui, China; 2 Engineering Research Center of Big Data Application in Private Health Medicine, Fujian Province University, Putian, Fujian, China; 3 Institutes of Physical Science and Information Technology, Anhui University, Hefei, Anhui, China

**Keywords:** Anticancer peptide, Deep learning, Handcrafted feature, Features fusion, Prediction

## Abstract

An emerging type of therapeutic agent, anticancer peptides (ACPs), has attracted attention because of its lower risk of toxic side effects. However process of identifying ACPs using experimental methods is both time-consuming and laborious. In this study, we developed a new and efficient algorithm that predicts ACPs by fusing multi-view features based on dual-channel deep neural network ensemble model. In the model, one channel used the convolutional neural network CNN to automatically extract the potential spatial features of a sequence. Another channel was used to process and extract more effective features from handcrafted features. Additionally, an effective feature fusion method was explored for the mutual fusion of different features. Finally, we adopted the neural network to predict ACPs based on the fusion features. The performance comparisons across the single and fusion features showed that the fusion of multi-view features could effectively improve the model’s predictive ability. Among these, the fusion of the features extracted by the CNN and composition of k-spaced amino acid group pairs achieved the best performance. To further validate the performance of our model, we compared it with other existing methods using two independent test sets. The results showed that our model’s area under curve was 0.90, which was higher than that of the other existing methods on the first test set and higher than most of the other existing methods on the second test set. The source code and datasets are available at https://github.com/wame-ng/DLFF-ACP.

## Introduction

Cancer is one of the leading causes of death worldwide (Xi et al., 2020; Yue et al., 2019). In 2018, a total of 9.6 million people died from cancer (Bray et al., 2018). The economic burden of cancer is also very high (Mattiuzzi & Lippi, 2019). Traditional cancer treatments, such as chemotherapy, radiotherapy, or hormone therapy, have varying risks of side effects for patients (Wang, Lei & Han, 2018; Yaghoubi et al., 2020). Additionally, the use of anticancer drugs is associated with drug resistance (Wijdeven et al., 2016). Therefore, it is necessary to develop new methods for cancer treatment. Compared with traditional antibiotics and chemotherapy, anticancer peptides (ACPs) have been shown to exhibit broad spectrum activity without the development of drug resistance (Huang et al., 2015). The toxicity of ACPs to cancer cells is mainly due to the electrostatic attraction between the positively-charged ACPs and the negatively-charged components of the cancer cells (Li & Nantasenamat, 2019; Tornesello et al., 2020). ACP-based drugs open up broader prospects for cancer treatment (Kuroda et al., 2015). Although they have many advantages, the accurate and prompt identification of ACPs remains a challenge.

Experimental methods that are currently used for the accurate identification of ACPs are difficult to use in high throughput screening because they are time-consuming and costly. Therefore, it is necessary to develop computational methods that can quickly and accurately identify ACPs. In the past decade, several proposed methods have used traditional machine learning to better identify ACPs. Tyagi et al. (2013) developed AntiCP, a predictor based on support vector machine (SVM), that used amino acid composition and binary profiles as feature descriptors. Hajisharifi et al. (2014) also developed a SVM-based model using Chou’s pseudo amino acid composition and the local-alignment based kernel. In order to more accurately predict ACPs, Vijayakumar & Lakshmi (2015) designed ACPP, a prediction tool based on the compositional information centroidal and distributional measures of amino acids. Using the optimal dipeptide combination, Chen et al. (2016) proposed a sequence-based predictor called iACP. Rao et al. (2020b) proposed a predictor called ACPred-Fuse that further improved feature ability by combining multi-view information. The multi-functional peptides prediction tools, ACPred-FL (Wei et al., 2018) and PEPred-Suite (Wei et al., 2019) could also be used to predict ACPs. In recent years, deep neural networks (DNN) achieved a good performance in bioinformatics (Le & Nguyen, 2019; Li et al., 2020; Yan et al., 2020), and some have been applied for ACP prediction. Yi et al. (2019) proposed a long short-term memory model to extract features from peptide sequences and predict ACPs. Yu et al. (2020) compared three different DNN architectures and found that the best model was based on bidirectional long short-term memory cells. Ahmed et al. (2020) constructed a new DNN architecture using parallel convolution groups to learn and combine three different features. Rao, Zhang & G (2020a) was the first to apply graph convolutional networks in ACP prediction. Liang et al. (2020) summarized and compared the existing ACP identification methods. Although the existing methods achieved some success, there was still room for improvement in their predictive abilities. Additionally, these methods usually use only handcrafted features or features extracted by DNN. Therefore, we hypothesized that if features extracted by the convolutional neural network (CNN) were fused with handcrafted features, the model could be more effective for predicting ACPs.

In this study, we developed a new prediction model: Deep Learning and Feature Fuse-based ACP prediction (DLFF-ACP). First, we used CNN channel to automatically extract the spatial features based on the peptide sequences. The most widely-used handcrafted features were then added to the inputs of the handcrafted feature channel for processing and the extraction of more effective features. Second, the features extracted by CNN channel with peptide sequences named CNN features were fused with the out of handcrafted feature channel and input to a classifier to predict the peptide class. CNN was more effective at considering spatial information (Su et al., 2020) and extracting important features from the sequences (Su et al., 2019), and adding handcrafted features could further improve the model’s sensitivity and identification of more sequence information. Finally, when compared with a model with a single feature, the model with fused features achieved better results, which confirmed the effectiveness of feature fusion when predicting performance.

## Materials & Methods

### Datasets

In this study, we selected datasets from ACPred-Fuse (Rao et al., 2020b) to develop and evaluate our model. The positive samples were collected from the studies of Chen et al. (2016) and Tyagi et al. (2013), as well as the ACPs database, CancerPPD (Tyagi et al., 2015). The negative samples were partly collected from Tyagi et al.’s (2013), and other negative sequences with no anticancer activity were selected from Swiss-Prot (Bairoch & Apweiler, 2000). To avoid classification bias, we used the CD-HIT program (Li & Godzik, 2006) to remove peptide sequences with a similarity greater than 0.8 in both the positive and negative datasets. Finally, the data set consisted of 332 positive samples and 2,878 negative samples. We randomly selected 250 positive samples and 250 negative samples for the training set. The remaining 82 positive samples and 2,628 negative samples were used as the test set. Because of the imbalance of positive and negative samples in the above test set, we also added a balanced test set (82 ACPs and 82 non-ACPs) from ACPred-FL (Wei et al., 2018) for performance comparison with other models. The datasets used in this study were most representative in the field of ACP identification, which was convenient for the comparison and analysis with other methods. The details of these datasets are shown in Table 1.

### Encoding

**Figure 1:**
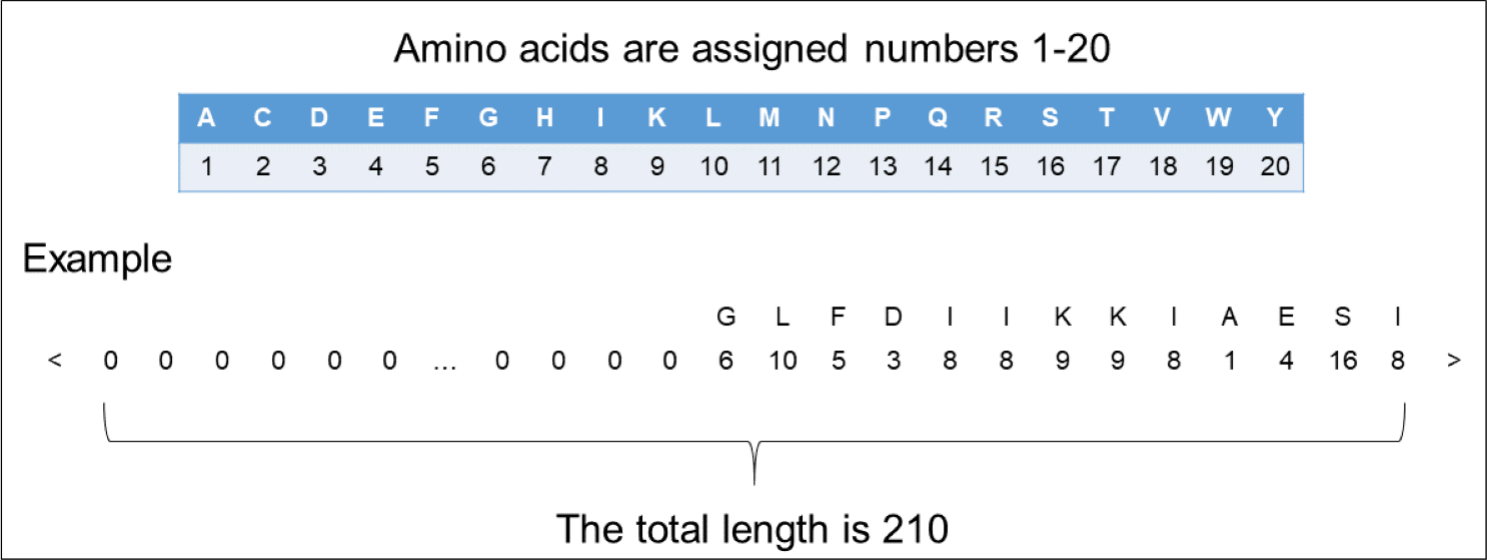
The representation of encoding peptide sequences.

In order to input the peptide sequences into the deep learning model, we needed to transform the format of the peptide sequences into numerical vector. We assigned a different number 1 to 20 to each of the 20 amino acids. Since the length of the peptide sequences input into the model should be fixed, we amplified the length of each peptide sequence to 210 by padding zero to fit our dataset’s longest ACP (207 amino acids) and non-ACP (96 amino acids). By tuning weights, the model quickly learned to ignore these padded zeros. The encoding process can be seen in Fig. 1.

### Handcrafted features

Different features can represent different information from the amino acid sequence. Here, we used three features that are commonly-used in ACP prediction: amino acid composition (AAC) (Bhasin & Raghava, 2004), dipeptide composition (DPC) (Saravanan & Gautham, 2015), and the composition of k-spaced amino acid group pairs (CKSAAGP) (Chen et al., 2018). In addition to these three feature representation methods, we also tested binary, physical and chemical properties (grouped amino acid composition and grouped di-peptide composition) and autocorrelation methods (Moran, Geary). However, they performed poorly in the model with remove the CNN channel, so we did not consider them in the final fused feature model. The feature representation of the peptide sequences was obtained through iFeature (Chen et al., 2018). The details of each feature are discussed in the following sections.

### AAC

AAC can be used to represent the frequency of each amino acid in the sequence. It is calculated using the following equation:

\begin{eqnarray*}f \left( a \right) = \frac{N \left( a \right) }{L} ,a\in \{ A,C,D,\ldots ,Y\} \end{eqnarray*}

**Table 1:** Summary of the two datasets.

Datasets	ACPred-Fuse’s dataset		ACPred-FL’s dataset	
	positive	negative	positive	negative
Training sets	250	250	250	250
Test sets	82	2628	82	82

where *f(a)* represents the frequency of the occurrence of amino acid type *a*, *N(a)* represents the total number of amino acids *a* appearing in the peptide sequences, and *L* represents the length of the peptide sequences.

### DPC

DPC is defined as the number of possible dipeptide combinations in a given peptide sequence. It can be calculated as:

\begin{eqnarray*}D \left( r,s \right) = \frac{{N}_{rs}}{L-1} ,r,s\in \{ A,C,D,\ldots ,Y\} \end{eqnarray*}

where *N*_*rs*_ is the number of dipeptides represented by amino acid types *r* and *s*.

### CKSAAGP

According to their physicochemical properties, the 20 amino acids can be divided into five classes (Lee et al., 2011). Table 2 lists the different physicochemical properties of these five classes and the amino acids contained in each class.

**Table 2:** Twenty amino acids were classified according to five physicochemical properties.

Physicochemical property	Amino acid
Aliphatic group (G1)	G, A, V, L, M, I
Aromatic group (G2)	F, Y, W
Positive charge group (G3)	K, R, H
Negative charge group (G4)	D, E
Uncharged group (G5)	S, T, C, P, N, Q

The CKSAAGP is used to calculate the frequency of amino acid group pairs separated by any k residues. Using *k* = 0 as an example, there are 25 0-spaced group pairs (i.e., G1G1, G1G2, G1G3, …, G5G5). The features can be defined as:

\begin{eqnarray*}{ \left( \frac{{N}_{G1G1}}{N} , \frac{{N}_{G1G2}}{N} , \frac{{N}_{G1G3}}{N} ,\ldots , \frac{{N}_{G5G5}}{N} \right) }_{25}. \end{eqnarray*}

Each value represents the number of times the residual group pair appears in the peptide sequence. For a peptide sequence with length *L*, when *k* = 0,1,2,3,4,5, the corresponding values of *N* are *L*-1, *L*-2, *L*-3, *L*-4, *L*-5, and *L*-6, respectively.

### Model architecture

**Figure 2:**
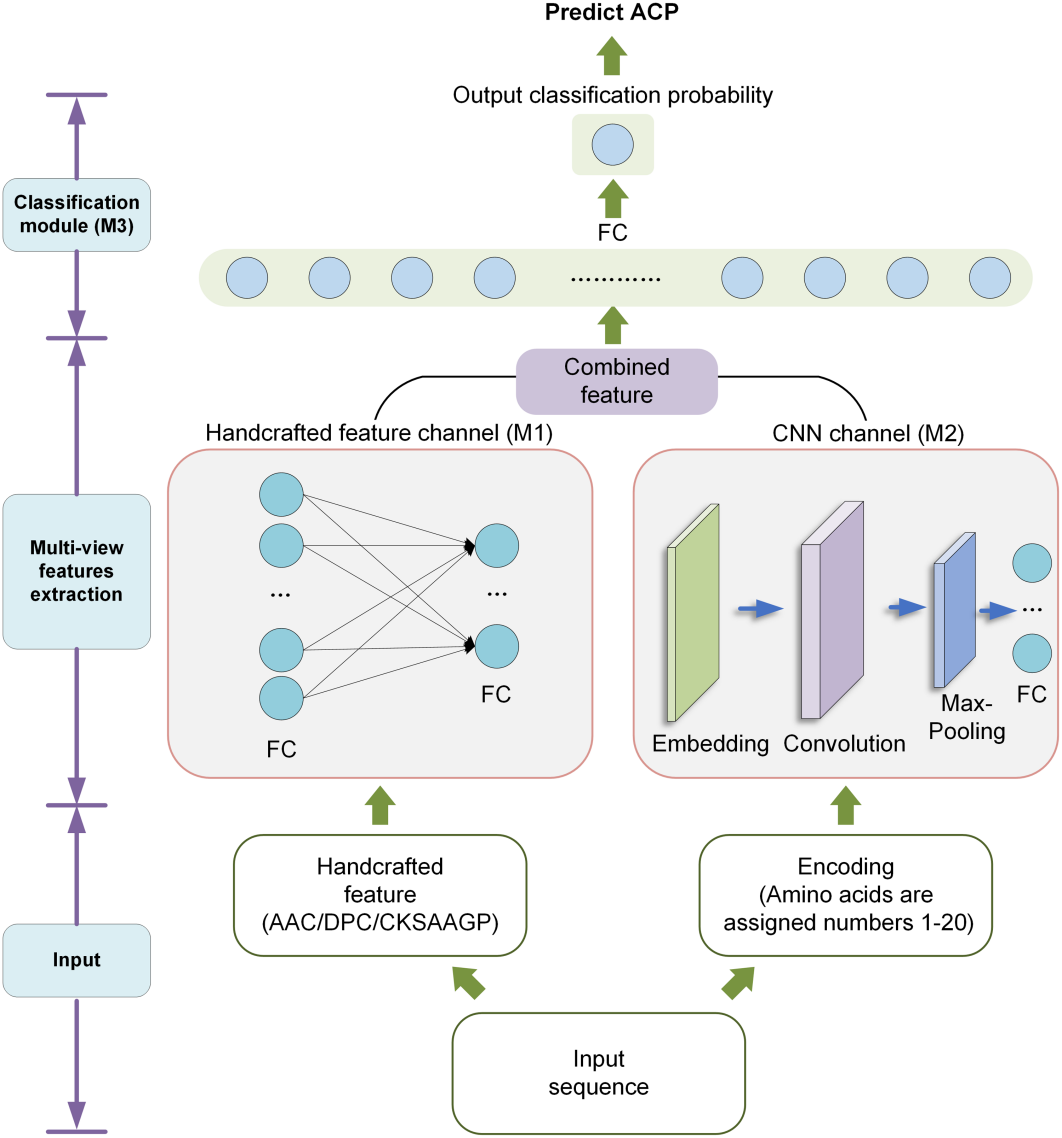
The architecture of DLFF-ACP.

In order to improve the recognition ACPs, we designed a new model called DLFF-ACP based on dual-channel DNN. We used Keras framework (Chollet, 2018) to build the model and TensorFlow (Abadi et al., 2016) deep learning library back-end. The DLFF-ACP model structure is shown in Fig. 2. It consists of three parts: the handcrafted feature channel (M1) for processing handcrafted feature information CNN channel (M2) for extracting features using CNN, and the classification module (M3) for classify the ACP and non-ACP based on the fused features. The sequence was converted into a numerical vector with a length of 210, processed by the embedding layer, and lastly, fed into CNN to extract features. Handcrafted features were fed into a network containing two full connection layers and then connected with the features extracted by CNN. Finally, this fused feature was used as the input of the M3 and output the probability value in the range [0, 1].

#### Handcrafted feature channel

In M1, the handcrafted features were input into a neural network with two full connection layers containing 128 and 64 units, respectively. To reduce overfitting, a dropout rate of 0.2 was used between the two full connection layers. The output of the module fused with the CNN features by concatenating during classification.

#### CNN channel

In M2, the encoded peptide sequences were input to automatically extract the spatial information features. The encoded peptide sequences were converted to a numeric vector length of 210. These numerical vectors were then input into the embedding layer, where discrete data was converted into fixed-size vectors. The embedded layer could express the corresponding relationship across discrete data. More importantly, the parameters of the embedded layer were constantly updated during the training process, which made the expression of the corresponding relationship even more accurate. We used a 1D convolutional (Conv1D) layer to automatically extract features. The convolutional layer had 32 kernels with a kernel size of 16. These convolutional layers were then fed into a max pooling layer with a kernel size of 8. The max pooling layer was used to reduce the number of parameters and overfitting. After that, the output of the max pooling layer was input to a fully connected layer containing 64 units.

#### Classification module

Finally, the output of the M1 was connected with the output of the M2 and served as the input of the module M3. This module consisted of a full connection layer with 64 units and an output layer with one unit. In the output layer, a probability value between 0 and 1 was finally obtained using sigmoid as the activation function. For these probability values, a value greater than 0.5 was considered ‘ACP’, and otherwise was considered ‘non-ACP’.

### Performance evaluation

To evaluate the performance, we adopted four metrics that are widely-used in machine learning for two-class prediction problems: sensitivity (SE), specificity (SP), accuracy (ACC) and Matthew’s correlation coefficient (MCC) (Chu et al., 2019; Le et al., 2020; Yue, Chu & Xia, 2020). The four metrics are defined as follows:

\begin{eqnarray*}SE& = \frac{TP}{TP+FN} \end{eqnarray*}

\begin{eqnarray*}SP& = \frac{TN}{TN+FP} \end{eqnarray*}

\begin{eqnarray*}ACC& = \frac{TP+TN}{TP+TN+FP+FN} \end{eqnarray*}

\begin{eqnarray*}MCC& = \frac{TP\times TN-FP\times FN}{\sqrt{(TP+FN)\times (TP+FP)\times (TN+FN)\times (TN+FP)}} \end{eqnarray*}

where TP, TN, FP, and FN represent the number of true positives (i.e., ACPs classified correctly as ACPs), true negatives (i.e., non-ACPs classified correctly as non-ACPs), false positives (i.e., ACPs classified incorrectly as non-ACPs), and false negatives (i.e., non-ACPs classified incorrectly as ACPs), respectively. In order to better measure the classifier’s overall performance, we also used area under curve (AUC) (Shi et al., 2019) as the threshold-independent evaluation metric. AUC is defined as the area bounded by the receiver operating characteristic (ROC) curve and coordinate axis. The closer the AUC is to 1.0, the higher the authenticity of the detection method (Bin et al., 2020; McDermott et al., 2019).

## Results

### Compositional analysis

**Figure 3:**
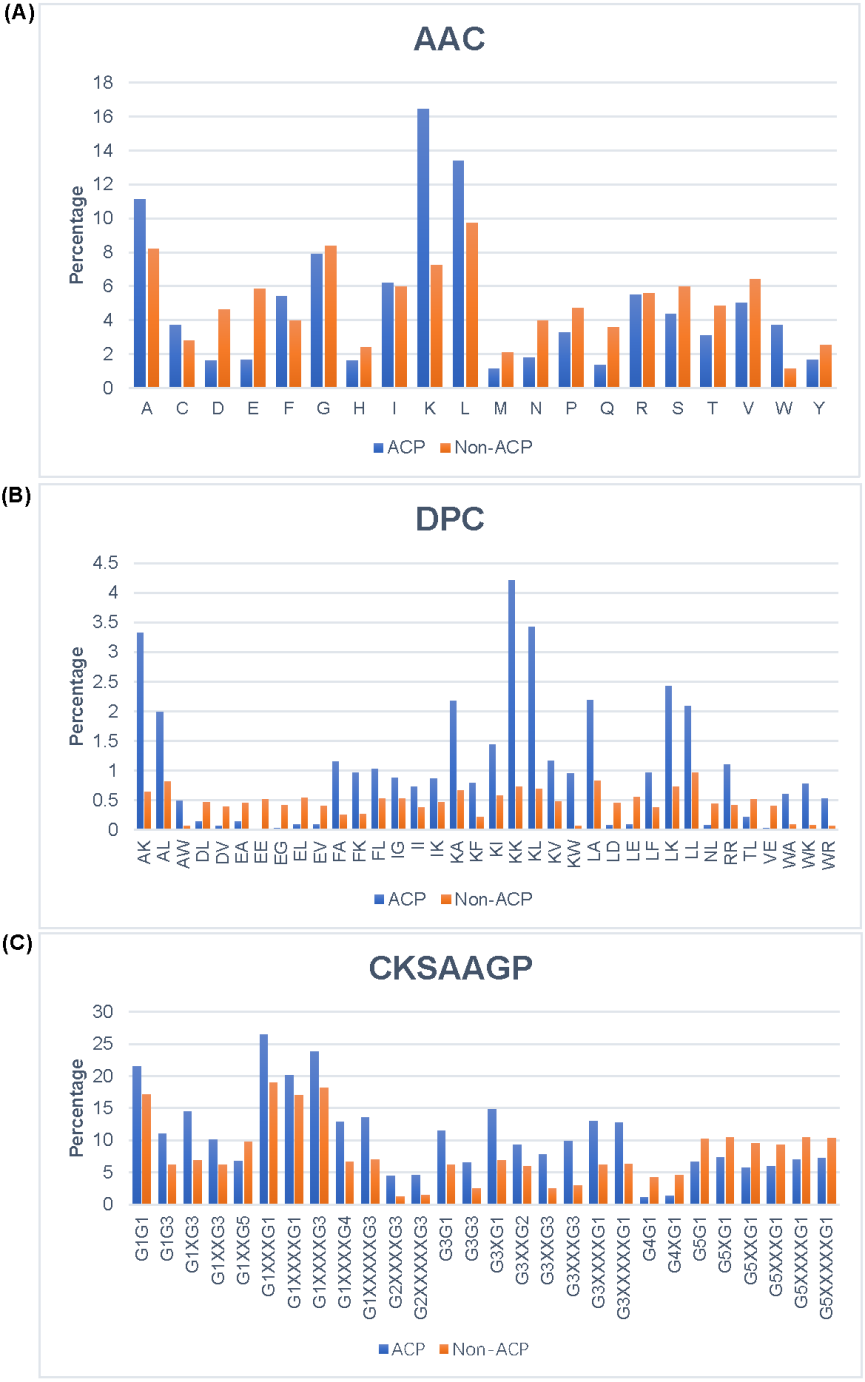
Analysis of different features between ACP and Non-ACP. (A) AAC. (B) DPC. (C) CKSAAGP.

In order to better understand the differences between ACPs and non-ACPs, we conducted different types of analyses on the total training and test datasets. The AAC analysis results Fig. 3A show that ACPs had a higher tendency to contain A, F, K, L, and W compared to non-ACPs, while D, E, N, Q, and S were more abundant in non-ACPs. This suggested that ACPs contain more positively-charged amino acids than non-ACPs. The selective toxicity of ACPs is also thought to be due to the close electrostatic interaction between positively-charged ACPs and negatively-charged cancer cells. The DPC analysis results Fig. 3B show that dipeptides AK, AL, FA, KA, KK, KL, KW, LA, LK, and LL were significantly more dominant in ACPs than in non-ACPs, and dipeptides DL, DV, EA, EE, EG, EL, EV, LD, LE, NL, TL, and VE were more abundant in non-ACPs than in ACPs. Additionally, the analysis results showed that 43% of dipeptides differed between ACPs and non-ACPS (*p* < 0.01). The CKSAAGP analysis results are shown in Fig. 3C, where X represents the interval between amino acids. As seen, the ratios of G1G1, G1G3, G1XG3 G1XXXG1, G1XXXXG1, G1XXXXG3, G1XXXXG4, G1XXXXXG3, G2XXXXG3, G2XXXXXG3, G3G1, G3G3, G3XG1, G3XXG2, G3XXG3, G3XXXG3, G3XXXXG1, G3XXXXXG1, G1G3, G1G1, and G1XXG3 were higher in the ACPs than in non-ACPs. However, G1XXG5, G4G1, G5XXXXXG1, G5XG1, G4XG1, G5XXXG1, G5XXXXG1, G5G1, and G5XXG1 ratios in ACPs were higher than those in non-ACPs. It is worth noting that this is highly similar to our AAC and DPC analysis results. Across 150 compositions of k-spaced amino acid pairs, 121 showed differences between ACPs and non-ACPS (*p* < 0.01). In conclusion, we chose these features as the input features of our proposed method due to the significant differences in the analyses of these components.

### Parameters of CNN

In our model, we used CNN to extract the spatial features of a sequence. In order to find the best hidden layer setting, we selected different number of filter layers. Since our training set was small 500 samples, we considered using at most two layers when selecting CNN architectures. Using deeper layers meant introducing too many parameters, which could potentially cause overfitting of the model. We chose three different filter sizes: 32,64, and 128. Table 3 shows the performance of 10-fold cross validation on the training set with different number of filter layers. The model with 32 filters achieved the best performance and the highest AUC value of 0.89. Therefore, we chose to use 32 filters when building our model.

### Channel comparisons

**Figure 4:**
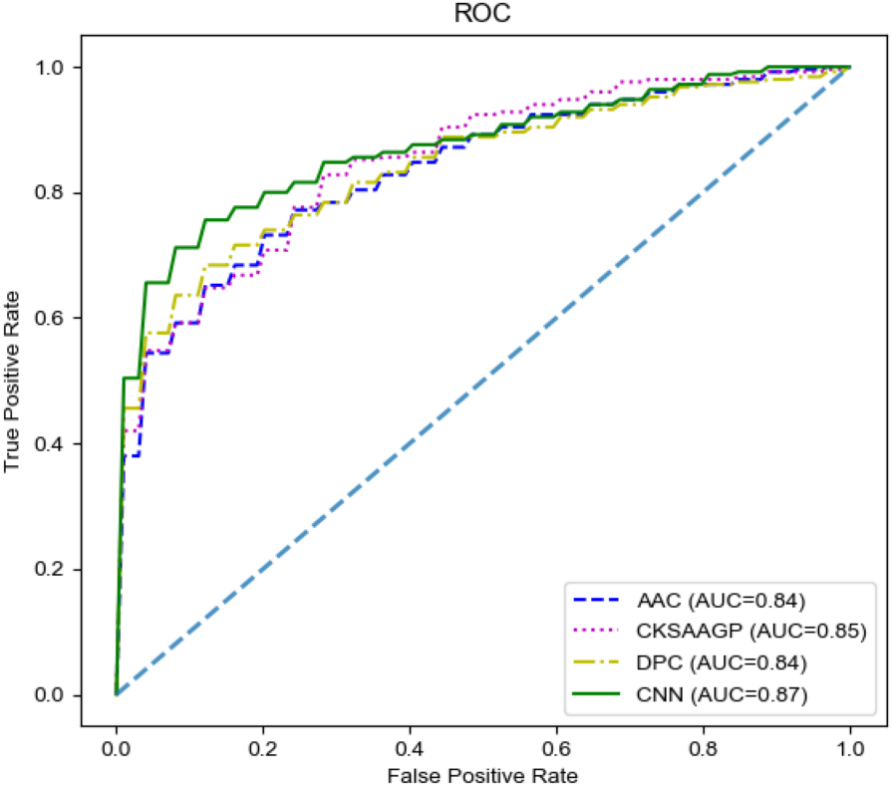
The ROC curves of different features on the training set.

In our model, the fusing feature information used for classification came from M1 and M2. To have a deeper understanding of the specific performance of these two channels, we compared M1 and M2. In M1, we chose three handcrafted features for comparison. Since there was only a single channel, we removed the model’s concatenate process. The output of a single channel was directly input into the M3 and the final prediction result was obtained. All the results were obtained through 10-fold cross-validation on the training set. The final comparison results are shown in Table 4 and Fig. 4. M2 achieved the highest SP (0.80), ACC (0.78), and MCC (0.57) values. Additionally, M2 still achieved the highest AUC. Therefore, the features extracted by the CNN channel achieved the best performance across the four different features.

### Comparison across different fusion features

**Figure 5:**
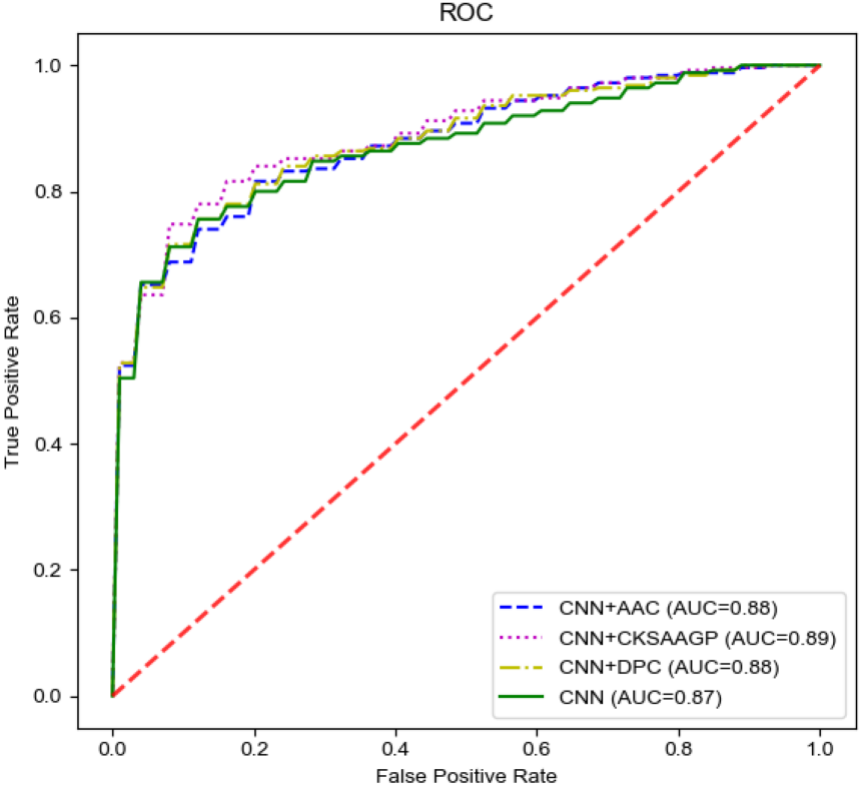
The ROC curves of different feature groups.

**Figure 6:**
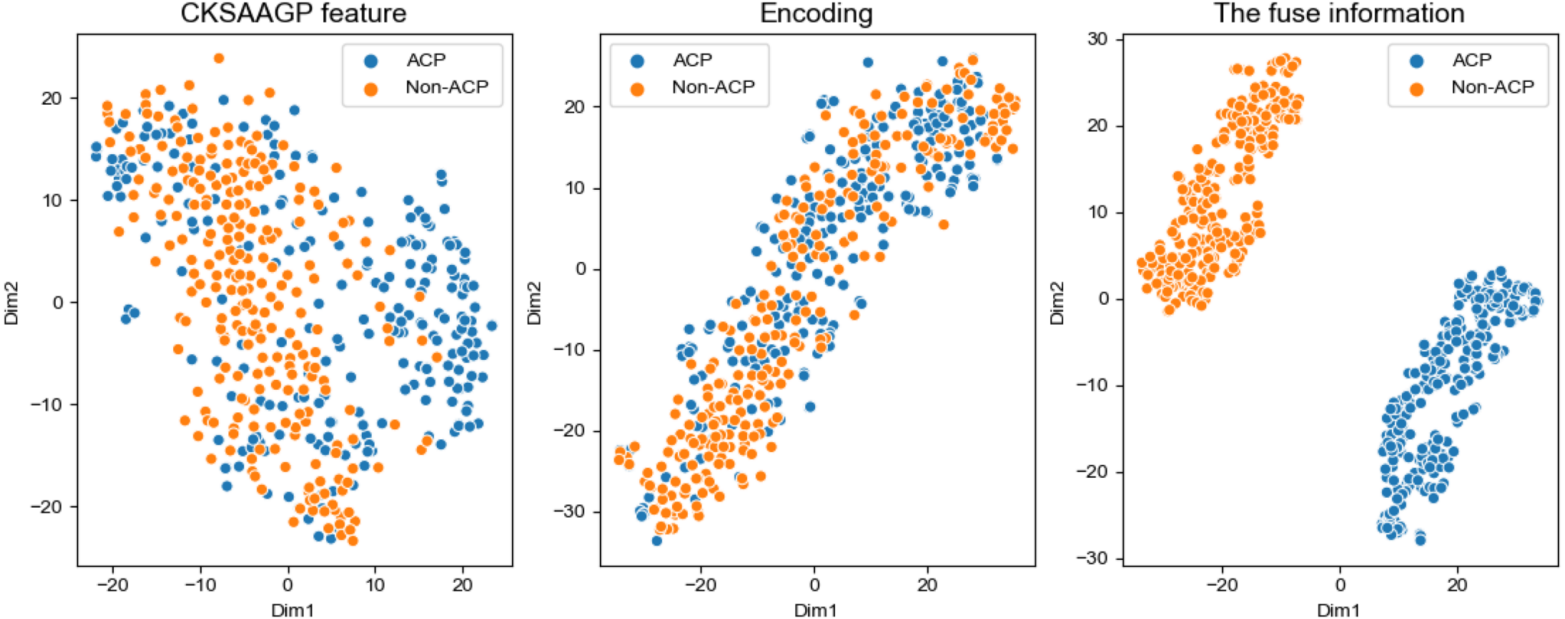
t-SNE distribution of different feature information. (A) Distribution of the CKSAAGP feature. (B) Distribution of the Encoding. (C) Distribution of the fusion of handcrafted feature channel and CNN channel.

To select the most effective feature fusion method with handcrafted features, we used CNN extracted features connected with three kinds of handcrafted features: AAC+CNN, CKSAAGP+CNN, and DPC+CNN. In order to verify whether the fused features were conducive to model performance, we compared the fused feature model with the model constructed using only the CNN channel (hereinafter referred to as the CNN model) with the best performance in individual feature models. Table 5 and Fig. 5 show the model performance across different scenarios in the training set. When compared with the CNN model only, the fused feature model achieved better performance and all the three fusion feature models were superior to the CNN model in performance. The CNN+AAC group had the highest SP (0.84) and the CNN+CKSAAGP group had the highest SE, ACC, and MCC, at 0.82, 0.82 and 0.65 respectively. When compared with the CNN model, the independent threshold evaluation method’s AUC of the fusion feature model also improved. To better understand the distribution of feature information, we used t-distributed stochastic neighbor embedding (t-SNE) (Maaten & Hinton, 2008) to visualize the feature information, and the results are shown in Fig. 6. Figure 6A shows the feature of CKSAAGP, Fig. 6B shows the sequence after encoding, and Fig. 6C shows the fusion of the output information of M1 and M2. The fused information can effectively differentiate ACPs from non-ACPs, fully demonstrating the effectiveness of using fusion features.

**Table 3:** Comparison of 10-fold cross validation results of different CNN architectures on the training data set.

Filters	ACC	Sen	Spe	MCC	AUC
32	**0.82**	**0.82**	**0.83**	**0.65**	**0.89**
64	0.80	0.79	0.79	0.60	0.88
32-64	0.78	0.76	0.80	0.57	0.87
64-128	0.78	0.78	0.78	0.56	0.87

**Notes.**

The highest values are highlighted in bold.

**Table 4:** Comparison of 10-fold cross validation results of different features on the training data set.

Channel	Features	SE	SP	Acc	MCC
M1	AAC	0.75	0.77	0.76	0.53
	CKSAAGP	0.74	0.78	0.76	0.53
	DPC	**0.76**	0.77	0.76	0.53
M2	CNN feature	**0.76**	**0.80**	**0.78**	**0.57**

**Notes.**

The highest values are highlighted in bold.

### Comparing the proposed model with existing methods

To verify the performance of our proposed model, we compared it with six traditional machine learning methods: AntiCP (Tyagi et al., 2013), Hajisharifi’s method (Hajisharifi et al., 2014), iACP (Chen et al., 2016), ACPredFL (Wei et al., 2018), PEPred-Suite (Wei et al., 2019), ACPred_Fuse (Rao et al., 2020b), and two deep learning methods, DeepACP (Yu et al., 2020) and ACP-MHCNN (Ahmed et al., 2020). We used the results of traditional machine learning method comparisons from the literature (Rao et al., 2020b). It should be noted that all models were used on the same training set, and the comparison results are shown in Table 6. Our model performed better in terms of SE, with an improvement of 0.05−0.28 compared with the other models. Our model’s MCC was similar to that of ACPred-Fuse and higher than those of the other models. However, our model’s AUC was higher than the other models. Our model was overall superior to the other models at distinguishing ACPs from non-ACPs. To further verify the performance of our model, we compared our method with other methods on the ACPred-FL’s dataset. To make a fair comparison, we used this training set to train all models, and tested the test dataset. The performance comparison results of all the models are illustrated in Table 7. Compared to AntiCP, Hajisharifi’s method, iACP, and DeepACP, our model showed an improvement of 0.04−0.13 and 0.08−0.25 in ACC and MCC, respectively. Although our model’s performance in this data set was a litter lower than that of ACPred-FL and ACP-MHCNN, it was generally better than most of the other methods.

**Table 5:** Performance comparison of different feature groups on the training set.

Feature group	SE	SP	ACC	MCC
CNN+AAC	0.74	**0.84**	0.79	0.59
CNN+CKSAAGP	**0.82**	0.83	**0.82**	**0.65**
CNN+DPC	0.80	0.79	0.80	0.60
CNN	0.76	0.80	0.78	0.57

**Notes.**

The highest values are highlighted in bold.

**Table 6:** Comparison with other existing methods on the test data set.

Methods	SE	SP	ACC	MCC	AUC
AntiCP_ACC	0.68	0.89	0.88	0.29	0.85
AntiCP_DC	0.68	0.83	0.82	0.22	0.83
Hajisharifi’s method	0.70	0.88	0.88	0.29	0.86
iACP	0.55	0.89	0.88	0.23	0.76
ACPred-FL	0.70	0.86	0.85	0.26	0.85
ACPred-Fuse	0.72	**0.90**	**0.89**	**0.32**	0.87
DeepACP	0.78	0.86	0.86	0.31	0.88
ACP-MHCNN	0.78	0.79	0.79	0.23	0.85
DLFF-ACP	** 0.83**	0.86	0.86	** 0.32**	** 0.90**

**Notes.**

The highest values are highlighted in bold.

**Table 7:** Comparison with other existing methods on the ACPred-FL’s data set.

Methods	SE	SP	ACC	MCC	AUC
AntiCP_ACC	0.68	0.87	0.77	0.56	0.83
AntiCP_DC	0.74	0.84	0.79	0.59	0.84
Hajisharifi’s method	0.67	0.87	0.77	0.55	0.82
iACP	0.68	0.80	0.74	0.49	0.80
ACPred-FL	0.81	** 0.96**	0.88	0.78	** 0.94**
DeepACP	0.89	0.77	0.83	0.66	0.87
ACP-MHCNN	**0.98**	0.84	**0.91**	**0.82**	0.93
DLFF-ACP	0.88	0.87	0.87	0.74	0.91

**Notes.**

The highest values are highlighted in bold.

## Discussion

In this study, we developed a new model for predicting ACPs based on deep learning and multi-view feature fusion. We integrated the handcrafted features into a deep learning framework and predicted the peptide class using a fully connected neural network. Different types of features relay different sequence information. CNN features focus on spatial information, while handcrafted features provide sequence composition information or physical and chemical properties. We compared the single channel model, and the results showed that the CNN features had better results. Additionally, we compared the fusion of different handcrafted features and CNN features, and we found that the fusion of CKSAAGP features and CNN features had the best performance. The fusion of these features enriched the final features and improved the performance of the model. We compared the model with a single channel, and the results showed that the dual-channel model could achieve better performance, which validated our hypothesis. To verify the robustness of our model, we also compared the performance of various models on the test set. In this test set, the number of negative samples was greater than the number of positive samples, which made it closer to the real data in practical application, and the results showed that our model performed better. To test the model’s generalization, we added a dataset with a balance of positive and negative samples in the test set, derived from ACPred-FL. The results showed that our performed better than most models.

## Conclusion

ACPs show many strengths in the treatment of cancer, but identifying ACPs with existing experimental methods is time consuming and laborious. In this study, we proposed a fast and efficient ACP predictive model based on dual-channel deep learning ensemble method. By fusing handcrafted features and features extracted by CNN, our model could effectively predict ACPs. Different comparative experiments confirmed that this model had excellent performance. In conclusion, the proposed predictor is more effective and promising for ACP identification and can be used as an alternative tool for predicting ACPs, especially in independent test sets that contain more negative samples. In future research, we will use different network architectures to find latent features, such as generative adversarial networks. Additionally, some methods that have been successfully used in natural language processing may also be considered.

## Additional Information and Declarations

### Data Availability

The following information was supplied regarding data availability: The data and code is available at GitHub: https://github.com/wame-ng/DLFF-ACP.
